# An intercountry comparison of the impact of the paediatric live attenuated influenza vaccine (LAIV) programme across the UK and the Republic of Ireland (ROI), 2010 to 2017

**DOI:** 10.1111/irv.13099

**Published:** 2023-02-13

**Authors:** Mary A. Sinnathamby, Fiona Warburton, Arlene J. Reynolds, Simon Cottrell, Mark O'Doherty, Lisa Domegan, Joan O'Donnell, Jillian Johnston, Ivelina Yonova, Suzanne Elgohari, Nicola L. Boddington, Nick Andrews, Joanna Ellis, Simon de Lusignan, Jim McMenamin, Richard G. Pebody

**Affiliations:** ^1^ UK Health Security Agency London UK; ^2^ Public Health Scotland Glasgow UK; ^3^ Public Health Wales Cardiff UK; ^4^ Public Health Agency Northern Ireland Belfast UK; ^5^ Health Service Executive‐Health Protection Surveillance Centre Dublin Ireland; ^6^ Royal College of General Practitioners (RCGP) Research and Surveillance Centre (RSC) London UK; ^7^ University of Surrey Guilford UK; ^8^ University of Oxford UK

**Keywords:** children, influenza, live attenuated vaccine, United Kingdom, vaccination

## Abstract

**Background:**

The universal paediatric live attenuated influenza vaccine (LAIV) programme commenced in the United Kingdom (UK) in 2013/2014. Since 2014/2015, all pre‐school and primary school children in Scotland and Northern Ireland have been offered the vaccine. England and Wales incrementally introduced the programme with additional school age cohorts being vaccinated each season. The Republic of Ireland (ROI) had no universal paediatric programme before 2017. We evaluated the potential population impact of vaccinating primary school‐aged children across the five countries up to the 2016/2017 influenza season.

**Methods:**

We compared rates of primary care influenza‐like illness (ILI) consultations, confirmed influenza intensive care unit (ICU) admissions, and all‐cause excess mortality using standardised methods. To further quantify the impact, a scoring system was developed where each weekly rate/z‐score was scored and summed across each influenza season according to the weekly respective threshold experienced in each country.

**Results:**

Results highlight ILI consultation rates in the four seasons' post‐programme, breached baseline thresholds once or not at all in Scotland and Northern Ireland; in three out of the four seasons in England and Wales; and in all four seasons in ROI. No differences were observed in the seasons' post‐programme introduction between countries in rates of ICU and excess mortality, although reductions in influenza‐related mortality were seen. The scoring system also reflected similar results overall.

**Conclusions:**

Findings of this study suggest that LAIV vaccination of primary school age children is associated with population‐level benefits, particularly in reducing infection incidence in primary care.

## 1 INTRODUCTION

Traditional seasonal influenza vaccination campaigns in the United Kingdom (UK) and the Republic of Ireland (ROI) routinely targeted those at higher risk of severe disease; those aged ≥65 year olds, individuals aged between 6 months and 64 years in a clinical risk group; and those pregnant women plus frontline healthcare workers to protect both themselves and their vulnerable patients.^1^, ^2^

In 2012, following recommendations by the UK Joint Committee on Vaccination and Immunisation (JCVI), the introduction of a universal paediatric influenza vaccination programme commenced with the ultimate vision to vaccinate all children aged 2 to 16 years of age in the UK through the incremental phased roll‐out of a newly licensed live attenuated influenza vaccine (LAIV).^3^ The programme was introduced based on the projected direct and indirect beneficial impact of vaccinating school age children on the wider population on reducing influenza transmission.^4^ Observational data on such indirect reductions are however limited to individual country assessments in the UK and elsewhere.^5^, ^6^, ^7^, ^8^, ^9^

The programme began in 2013/2014 with all countries of the UK initially offering vaccine to all children aged 2 to 3 years with Wales additionally offering the vaccine to children of School Year 7 age. In England, a series of geographically discrete pilot areas offered influenza vaccine to all children of primary school age. The following year in 2014/2015, the programme was further extended to include all children aged 2 to 4 years across the UK, with England continuing its pilot programme and Wales continuing to offer LAIV to all children aged 2 to 4 years and those at School Year 7 age. Scotland and Northern Ireland, on the other hand, started vaccinating all primary school‐aged children (5 to 11 years) in 2014/2015. In 2015/2016, the programme was extended to all children of School Years 1 and 2 in England and Wales (where the offer of LAIV to children of School Year 7 age was withdrawn); this was further extended to include all children of School Year 3 in 2016/2017. Over this same period, the vaccination of all primary school age children continued in Scotland and Northern Ireland. Uptake of the LAIV vaccine amongst primary school age children, besides involving additional age cohorts, has been higher in Scotland and Northern Ireland (73.0% and 78.3%, respectively, in children aged 4–11 years) in 2016/2017 compared with England and Wales (55.4% in children aged 5–8 years and 66.9% in children aged 4–7 years, respectively) in 2016/2017.^10^

During the period 2013/2014 to 2016/2017, the ROI had not yet introduced the universal paediatric influenza vaccination; the influenza vaccine programme included high‐risk groups.

This differential roll‐out of the paediatric programme across these five countries provided a unique opportunity to compare the indirect and overall effects of vaccinating children on the epidemiology of influenza across the UK and ROI across several seasons.

A number of approaches have been developed over the years to standardise the monitoring of influenza surveillance across countries, in particular the World Health Organization Pandemic Influenza Severity Assessment (WHO PISA) initiative, which looks to describe influenza activity and impact and to use this to inform national and global risk assessments more uniformly.^11^ The present study aims to use established methods to determine potential differences in the impact of the paediatric influenza vaccination programmes on influenza between countries of the UK and the ROI.

This study assesses the overall and indirect impact of vaccinating primary school age children with influenza vaccine by comparing the epidemiology of influenza in the UK and ROI where varying vaccine strategies were implemented using a range of primary care, secondary care and mortality indicators.

## 2 METHODS

**FIGURE 1:**
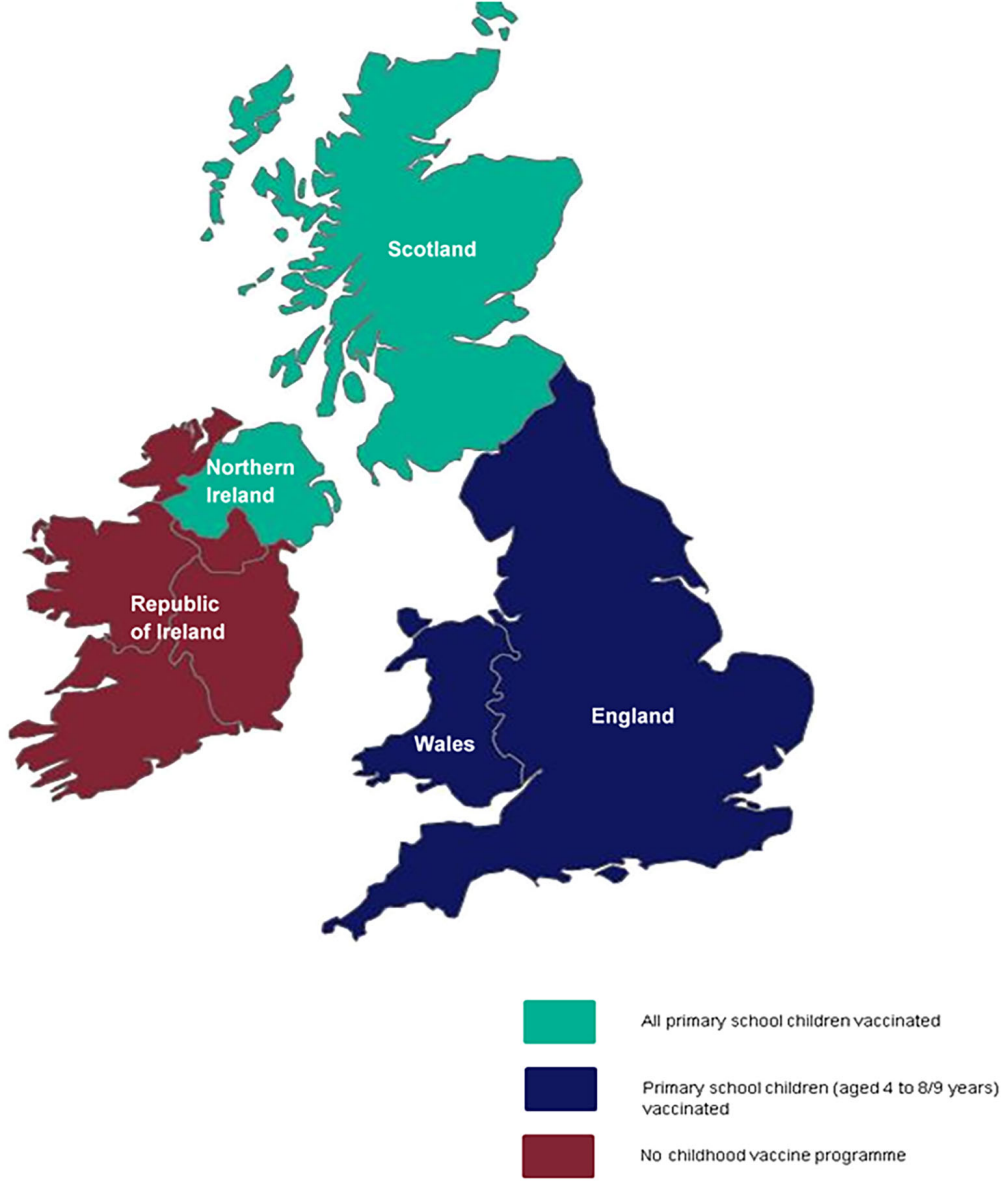
Map of the UK and Republic of Ireland (ROI) representing the different paediatric influenza vaccination schemes.

Countries of the UK and the ROI were categorised according to the delivery method of their respective paediatric vaccination programme (Figure 1).

The study periods were defined as the pre‐programme (pre‐vaccine) period including seasons between 2010/2011 and 2012/2013 and the post‐programme (post‐introduction of the vaccine) period including seasons between 2013/2014 and 2016/2017.

The following WHO PISA surveillance indicators were used to assess the activity and impact of the programme in all five countries^11^:

1. The activity indicator used in primary care was general practice (GP) influenza‐like illness (ILI) consultation rates.
2. The impact indicator used in secondary care was intensive care unit/high‐dependency unit (ICU/HDU) admission rates.
3. The mortality indicators used were all‐cause and influenza‐attributable excess mortality.

All data were categorised and analysed in the following age categories: all ages, less than 15 year olds, 15–64 year olds and ≥65 year olds.

### 2.1 Statistical methods

#### 2.1.1 Moving epidemic method model

For primary and secondary care indicators, the moving epidemic method (MEM) model was used to calculate five standardised thresholds (baseline, low, moderate, high and very high) for each respective country to enable comparison between countries. This method has been well‐established to standardise surveillance outputs, particularly in Europe.^12^ Comparisons were made based on the 2016/2017 season's MEM thresholds using the available data from each country (Tables 1 and 2).

**TABLE 1:** Comparison of (a) GP ILI consultations schemes and (b) 2016/2017 MEM thresholds (overall and by age groups) for individual UK countries and ROI.

(a)					
	Health Protection Scotland	Public Health Agency Northern Ireland	RCGP (England)	Public Health Wales	HSE (Ireland)
Influenza illness definition recorded	ILI	Influenza and ILI	ILI	ILI	ILI
Historical data used for MEM	10 seasons (2005/06 to 2015/16, excl. 2009/10)	10 seasons (2005/06 to 2015/16, excl. 2009/10)	10 seasons (2005/06 to 2015/16, excl. 2009/10)	6 seasons (2010/11 to 2015/16, excl. 2009/10)	10 seasons (2005/06 to 2015/16, excl. 2009/10)
Proportion of population covered	98% (all GP practices)	98% (all GP practices)	2% (sentinel network of ~200 GP practices)	100% (all GP practices)	Sentinel network (ICGP) of around 60 GP practices

(b)						
GP ILI MEM thresholds, 2016/2017		Scotland *	Northern Ireland *	England *	Wales *	ROI *
All ages	Baseline threshold	<36.1	<47.9	<14.3	<10.7	<17.7
	Low	36.1 to <51.2	47.9 to <66.3	14.3 to <26.2	10.7 to <15.6	17.7 to <58.7
	Moderate	51.2 to <235.6	66.3 to <193.6	26.2 to <73.5	15.6 to <43.8	58.7 to <113.2
	High	235.6 to <462.6	193.6 to <310.9	73.5 to <116.1	43.8 to <69.2	113.2 to <151.3
	Very high	462.6+	310.9+	116.1+	69.2+	151.3+
<15 years	Baseline threshold	<10.6	<25.4	<11.0	<6.7	<16.8
	Low	10.6 to <14.5	25.4 to <34.0	11.0 to <16.4	6.7 to <10.5	16.8 to <54.7
	Moderate	14.5 to <68.4	34.0 to <107.6	16.4 to <51.9	10.5 to <34.0	54.7 to <130.3
	High	68.4 to <136.0	107.6 to <179.1	51.9 to <86.3	34.0 to <57.2	130.3 to <191.1
	Very high	136.0+	179.1+	86.3+	57.2+	191.1+
15–64 years	Baseline threshold	<20.4	<27.9	<14.5	<10.4	<20.1
	Low	20.4 to <34.5	27.9 to <50.3	14.5 to <25.0	10.4 to <17.2	20.1 to <53.4
	Moderate	34.5 to <112.9	50.3 to <131.0	25.0 to <63.9	17.2 to <52.0	53.4 to <120.1
	High	112.9 to <190.6	131.0 to <200.0	63.9 to <96.7	52.0 to <84.9	120.1 to <171.9
	Very high	190.6+	200.0+	96.7+	84.9+	171.9+
65 + years	Baseline threshold	<15.3	<28.2	<11.5	<7.4	<17.7
	Low	15.3 to <21.2	28.2 to <36.8	11.5 to <13.6	7.4 to <9.2	17.7 to <30.7
	Moderate	21.2 to <48.8	36.8 to <65.4	13.6 to <29.4	9.2 to <22.9	30.7 to <59.6
	High	48.8 to <70.5	65.4 to <84.3	29.4 to <41.3	22.9 to <34.2	59.6 to <80.0
	Very high	70.5+	84.3+	41.3+	34.2+	80.0+

Abbreviations: GP, general practice; ILI, influenza‐like illness; MEM, moving epidemic method; ROI, Republic of Ireland.

* Thresholds for <15 years, 15–64 years and 65 + years for Scotland, Northern Ireland, England, Wales and ROI are based on 6 seasons' data.

**TABLE 2:** Comparison of (a) ICU/HDU schemes and (b) 2016/2017 MEM thresholds (overall and by age groups) for individual UK countries and ROI.

(a)					
	Health Protection Scotland	Public Health Agency Northern Ireland	USISS (England)	Public Health Wales	HSE (Ireland)
Admission type recorded	ICU admissions only	ICU/HDU admissions	ICU/HDU admissions	ICU admissions only	ICU admissions only
Historical data used for MEM	5 seasons (2011/12 to 2015/2016)	5 seasons (2011/2012 to 2015/2016)	5 seasons (2011/2012 to 2015/2016)	5 seasons (2011/2012 to 2015/2016)	5 seasons (2011/2012 to 2015/2016)
Proportion of population covered	100% (ONS population estimates)	100% (ONS population estimates)	100% (ONS population estimates)	100% (ONS population estimates)	100% (CSO population estimates)

(b)						
ICU/HDU MEM thresholds, 2016/2017		Scotland *	Northern Ireland *	England	Wales✝	ROI
All ages	Baseline threshold	<0.04	<0.10	<0.06	<0.09	<0.03
	Low	0.04 to <0.12	0.10 to <0.27	0.06 to <0.10	0.09 to <0.11	0.03 to <0.10
	Moderate	0.12 to <0.41	0.27 to <0.51	0.10 to <0.31	0.11 to <0.37	0.10 to <0.33
	High	0.41 to <0.71	0.51 to <0.64	0.31 to <0.52	0.37 to <0.50	0.33 to <0.56
	Very high	0.71+	0.64+	0.52+	0.50+	0.56+
<15 years	Baseline threshold	<0.09		<0.07		<0.10
	Low	0.09 to <0.15		0.07 to <0.11		0.10 to <0.15
	Moderate	0.15 to <0.36		0.11 to <0.27		0.15 to <0.36
	High	0.36 to <0.46		0.27 to <0.41		0.36 to <0.47
	Very high	0.46+		0.41+		0.47+
15–64 years	Baseline threshold	<0.05		<0.05		<0.03
	Low	0.05 to <0.13		0.05 to <0.08		0.03 to <0.10
	Moderate	0.13 to <0.28		0.08 to <0.30		0.10 to <0.29
	High	0.28 to <0.36		0.30 to <0.52		0.29 to <0.38
	Very high	0.36+		0.52+		0.38+
65 + years	Baseline threshold	<0.06		<0.07		<0.14
	Low	0.06 to <0.26		0.07 to <0.14		0.14 to <0.36
	Moderate	0.26 to <0.59		0.14 to <0.44		0.36 to <0.87
	High	0.59 to <0.76		0.44 to <0.72		0.87 to <1.14
	Very high	0.76+		0.72+		1.14+

Abbreviations: CSO, Central Statistics Office; HDU, high‐dependency unit; ICU, intensive care unit; MEM, moving epidemic method; ONS, Office for National Statistics; ROI, Republic of Ireland.

* Shaded cells in the table represent age groups for which MEM thresholds could not be calculated, due to small numbers.

All MEM threshold calculations were carried out using the MEM model package in R version 4.3.3.^13^

#### 2.1.2 EuroMOMO and FluMOMO models

Mortality indicators were assessed using empirical thresholds based on the analysis of the EuroMOMO algorithm outputs; where the baseline threshold was defined as <2 z‐score, the low threshold as 2 to <6 z‐score, the moderate thresholds as 6 to <10 z‐score, the high threshold as 10 to 16 z‐score and the very high threshold as >16 z‐score. The same threshold values were applied to all age groups. The EuroMOMO model aims to provide weekly excess all‐cause mortality estimates using a time series Poisson regression model whilst taking into account trends, seasonal variation and corrections for delays.^14^ Z‐scores from the model outputs were used to determine thresholds. Thresholds were applied to the weekly excess estimates for comparisons across countries.

Influenza‐attributable mortality rates were also calculated using the FluMOMO algorithm, a multiplicative Poisson regression time series model with overdispersion.^15^, ^16^ The FluMOMO algorithm was run over six seasons (2011/2012 to 2016/2017).

All mortality models (EuroMOMO and FluMOMO version 4.2) were run in STATA 13.

#### 2.1.3 Scoring system

**FIGURE 2:**
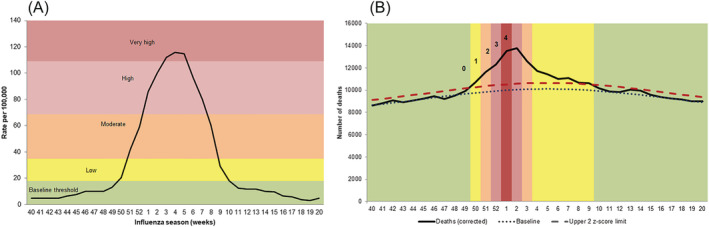
Scoring system for (A) primary care and secondary care indicator; and for (B) mortality indicator.

To further quantify the impact, a scoring system was set up where each weekly rate/z‐score was scored and summed across each season (from Week 40 to 20) according to the weekly threshold experienced. The scores for each threshold band were assigned as follows: 0 = *below baseline*; 1 = *low threshold*; 2 = *medium threshold*; 3 = *high threshold*; and 4 = *very high threshold* (Figure 2). Scores for the pre‐programme period were averaged from the overall season scores between 2010/2011 and 2012/2013 seasons for the primary care indicator and between 2011/2012 and 2012/2013 seasons for secondary care and mortality indicators. Scores for the post‐programme period were averaged from the overall season scores between 2013/2014 and 2016/2017 seasons.

### 2.2 Data sources

#### 2.2.1 Primary care indicator

GP influenza/ILI consultation rates per 100,000 were collected weekly from the 2010/2011 to the 2016/2017 seasons via automated/semiautomated extractions from GP systems, from the following previously described primary care schemes in the UK: the Oxford Royal College of General Practitioners (RCGP) Research and Surveillance Centre (RSC) for England, Health Protection Scotland, Public Health Agency Northern Ireland, Public Health Wales and Health Service Executive‐Health Protection Surveillance Centre (HSE‐HPSC) in Ireland.^17^, ^18^, ^19^, ^20^

MEM thresholds for the 2016/2017 season were calculated based on the availability of the data in each country and applied across all historical data (Table 1).

#### 2.2.2 Secondary care indicator

Weekly laboratory‐confirmed influenza admissions to ICU/HDU were collated from 2011/2012 to 2016/2017 from each country using their respective ICU surveillance systems. ICU/HDU admission rates were then calculated using each country's annual population data available from the Office for National Statistics (ONS) for the countries of the UK and from the Central Statistics Office (CSO) for the ROI.^21^, ^22^

MEM thresholds for the 2016/2017 season were calculated based on the availability of the data in each of the countries and applied across all historical data (Table 2).

#### 2.2.3 Mortality indicator

The national weekly number of all‐cause death registrations from week 40,2011 up to week 202,017 was collected for each country and computed into the EuroMOMO model to produce excess all‐cause mortality estimates in the form of z‐scores.

To determine influenza‐attributable deaths, in addition to the weekly number of all‐cause death registrations, weekly influenza activity (IA = influenza/ILI rate (per 100,000 population) × overall positivity (%)) and extreme temperature data for each country were inputted into the FluMOMO model.^15^, ^16^

## 3 RESULTS

Overall, notable differences were observed between countries that offered the LAIV programme to all primary school‐aged children (Scotland and Northern Ireland) during part of the post‐programme period (2014/2015 to 2016/2017) compared to those who incrementally introduced the programme (England and Wales) or who did not have a universal paediatric programme (ROI).

### 3.1 Primary care indicator

In the pre‐programme period, GP ILI consultation rates breached baseline thresholds in at least two of the three seasons in all countries except for Northern Ireland, which only breached its baseline threshold in one of the three seasons.

**FIGURE 3:**
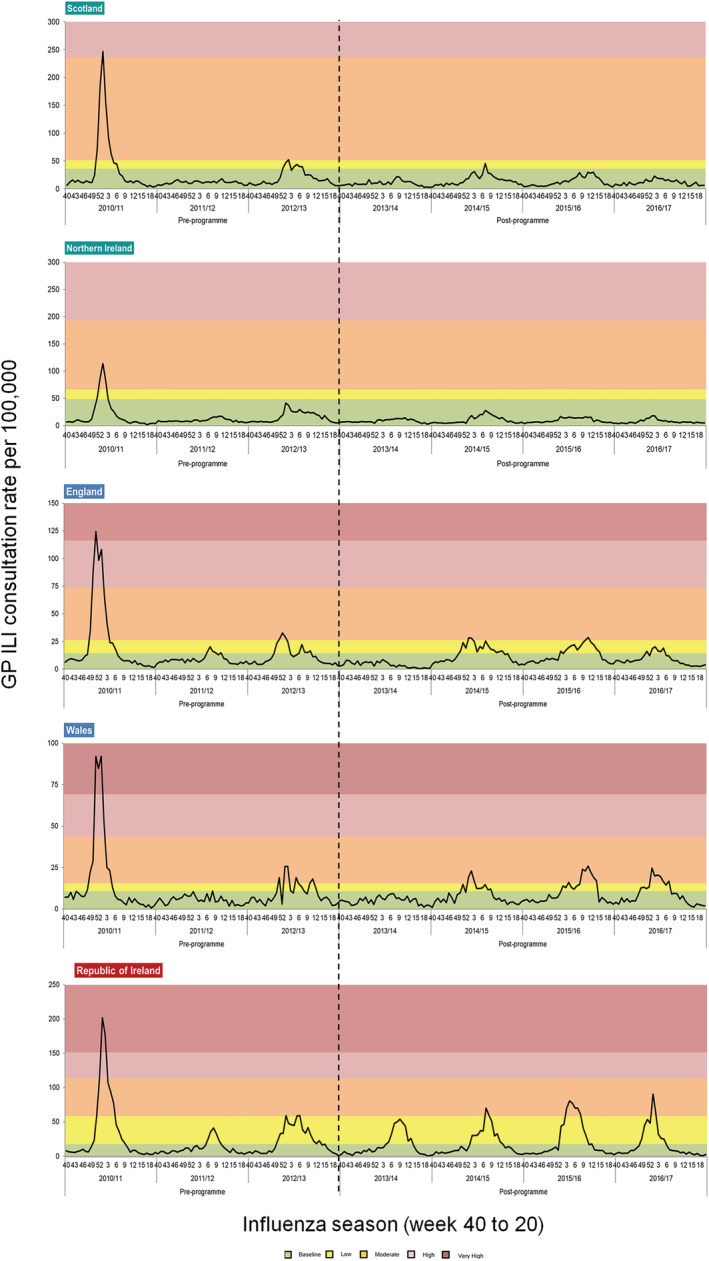
GP influenza‐like illness (ILI) consultations rates for all ages from 2010/2011 to 2016/2017 with individual country specific 2016/2017 Moving Epidemic Method (MEM) thresholds applied, UK and Republic of Ireland (ROI).

GP ILI consultations rates were evidently higher in the 2010/2011 season (a season following the 2009 influenza pandemic) across all countries; with all but Northern Ireland breaching the very high threshold for at least one week. In the 2011/2012 season, Scotland, Northern Ireland and Wales did not breach their baseline thresholds whereas England and the ROI breached their baseline thresholds for five consecutive weeks, respectively. A similar observation to that of the 2010/2011 season was noted in the pre‐programme season 2012/2013, with all countries but Northern Ireland breaching their baseline threshold (Figure 3).

Post‐programme observations showed evidence of lower overall (all ages) rates of GP ILI consultations in Scotland and Northern Ireland, which offered the LAIV vaccine to all primary school age children compared to the rates observed in England, Wales and ROI (Figure 3).

Scotland only breached their respective baseline threshold in one out of the four post‐programme seasons (in 2014/2015 for 1 week), and Northern Ireland did not breach their baseline threshold throughout all four post‐programme seasons. England and Wales breached their respective baseline thresholds in three out of four post‐programme seasons, including in 16 and 10 weeks of 2014/2015, in 14 and 13 weeks of 2015/2016 and in 6 and 11 weeks of 2016/2017, respectively. The ROI breached their baseline threshold in all four post‐programme seasons, for an average of 10 weeks in each post‐programme season.

Reductions in percentage change between the average pre‐ and post‐programme scores were seen overall (all ages) and in <15 year olds across all countries. The greatest overall (all ages) reductions were noted in Scotland and Northern Ireland countries vaccinating all primary school age children (reductions of 97% and 100%, respectively, in comparison to a range of 11%–25% in England, Wales and ROI). A similar observation was noted for <15 year olds, where Scotland and Northern Ireland saw reductions of 89% and 100%, respectively, and reductions of 53%, 38% and 33% were noted for England, Wales and ROI (Table 3).

**TABLE 3:** GP ILI consultations average scores for all ages, less than 15 year olds, 15–64 years olds and 65 + years for pre‐ (from 2010/2011 to 2012/2013) and post‐ (2013/2014 to 2016/2017) programme seasons, UK and ROI.

Score based on the sum of weekly (week 40 to 20) thresholds across pre‐ and post‐ programme seasons	All ages			<15 years			15–64 years			65 + years		
	Average pre‐programme season score	Average post‐programme season score	% change in average pre‐ and post‐programme score	Average pre‐programme season score	Average post‐programme season score	% change in average pre‐ and post‐programme score	Average pre‐programme season score	Average post‐programme season score	% change in average pre‐ and post‐programme score	Average pre‐programme season score	Average post‐programme season score	% change in average pre‐ and post‐programme score
Scotland	7.7	0.3	⇩97%	18.7	2.0	⇩89%	17.3	8.8	⇩50%	12.0	7.8	⇩35%
Northern Ireland	2.3	0.0	⇩100%	5.7	0.0	⇩100%	6.0	0.3	⇩96%	2.3	0.5	⇩79%
England	13.3	10.0	⇩25%	16.3	7.8	⇩53%	16.7	14.5	⇩13%	10.7	10.8	⇧1%
Wales	14.0	12.5	⇩11%	13.3	8.3	⇩38%	14.0	14.5	⇧4%	7.7	11.0	⇧43%
Republic of Ireland	16.0	12.5	⇩22%	16.0	10.8	⇩33%	16.3	14.5	⇩11%	8.3	16.3	⇧95%

Abbreviations: GP, general practice; ILI, influenza‐like illness; ROI, Republic of Ireland.

Reductions in percentage changes of the average scores between pre‐and post‐ programme scores were also noted in the 15 to 64 year olds in Scotland, Northern Ireland, England and ROI but not in Wales. Reductions in comparison to pre‐ and post‐programme average scores were less observed in older adults. In those aged 65 years and older, Scotland and Northern Ireland saw reductions (35% and 79%, respectively), whereas England, Wales and ROI saw increases in percentage change between the average pre‐ and post‐programme scores, with ROI seeing the highest increase of 95% (Table 3).

### 3.2 Secondary care indicators

Differences between vaccinating and non‐vaccinating countries in the post‐programme vaccination period compared with the pre‐programme introduction period were less notable through the ICU/HDU admission surveillance systems.

**FIGURE 4:**
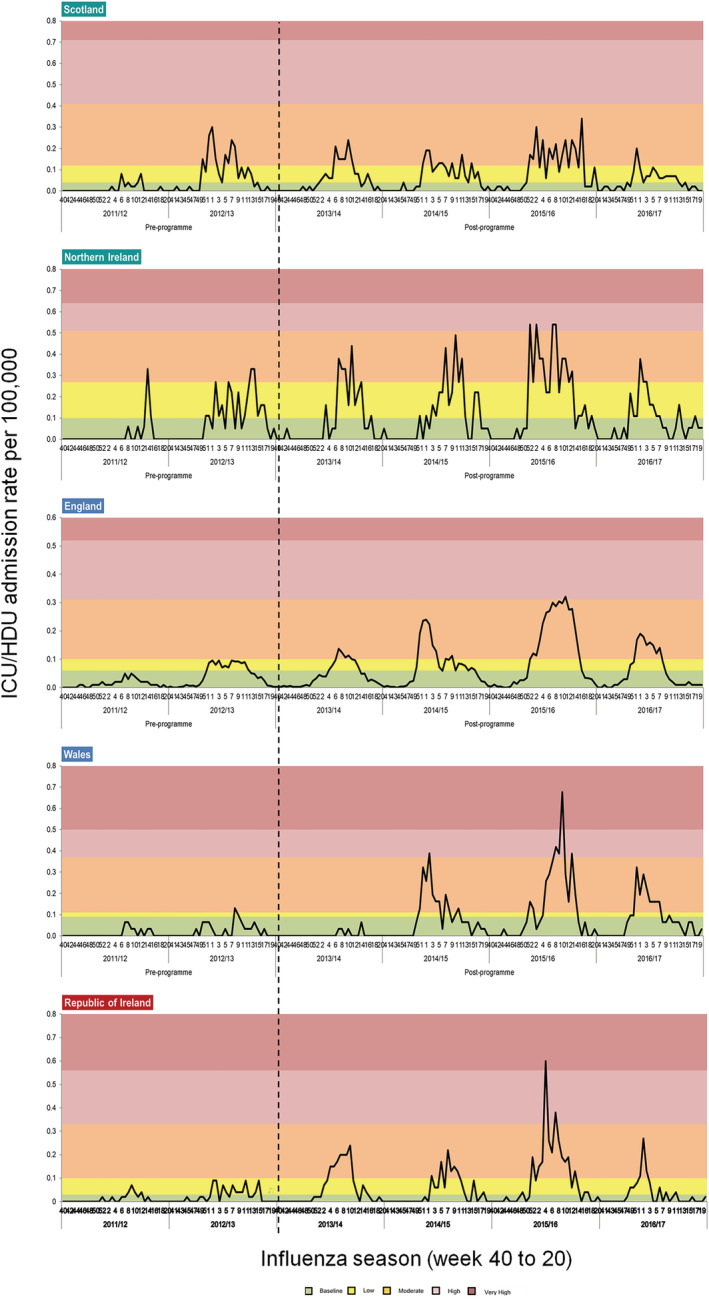
Intensive care unit (ICU)/high‐dependency unit (HDU) admission rates for all ages from 2011/2012 to 2016/2017 with individual country specific 2016/2017 Moving Epidemic Method (MEM) thresholds applied, UK and Republic of Ireland (ROI).

During the pre‐programme period, Scotland and Northern Ireland experienced higher ICU/HDU admission rates than England, Wales and ROI, breaching their baseline threshold in two out of two seasons (2011/2012 and 2012/2013) (Figure 4). Medium threshold levels were also breached in 1 week (at 0.33 per 100,000) in the 2011/2012 season for Northern Ireland and for 2 (peaking at 0.33 per 100,000) and 8 weeks (peaking at 0.30 per 100,000) in the 2012/2013 season for Northern Ireland and Scotland, respectively.

This compares to England and Wales only breaching their respective baseline threshold in one out two seasons, for 10 weeks (peaking at 0.10 per 100,000) and 1 week (at 0.13 per 100,000), respectively, in the 2012/2013 season. ROI breached their baseline threshold in both pre‐programme seasons, for 4 (peaking at 0.07 per 100,000) and 15 weeks (peaking at 0.09 per 100,000) in the 2011/2012 and 2012/2013 seasons, respectively.

During the post‐programme period, all countries breached their respective baseline to medium thresholds in all four out of four seasons, except for Wales who did not breach its baseline threshold in the 2013/2014 season and therefore only breached their baseline threshold for three out of four seasons. All countries experienced greater ICU/HDU admission rates in the 2015/2016 season compared to the other post‐programme seasons, with all countries breaching their medium threshold for at least 11 weeks (range 11–16 weeks) in this season. Wales and ROI also reached their respective very high threshold during the 2015/2016 season.

No overall reductions in percentage change were observed through the scoring system between pre‐ and post‐ programme scores in all countries (Table 4). A smaller increase in percentage change between the average pre‐ and post‐programme scores was noted in the vaccinating countries (Scotland and Northern Ireland—66% and 86% increase, respectively) in comparison with countries with an incremental roll‐out (England and Wales—209% and 1083% increases, respectively) and no universal programme (ROI—183% increase) (Table 4).

**TABLE 4:** ICU/HDU admission average scores for all ages, less than 15 year olds, 15–64 years olds and 65 + years olds for pre‐ (from 2011/2012 to 2012/2013) and post‐ (2013/2014 to 2016/2017) programme seasons, UK and ROI.

Score based on the sum of weekly (week 40 to 20) thresholds across pre‐ and post‐ programme seasons	All ages			<15 years			15–64 years			65 + years		
	Average pre‐programme season score	Average post‐programme season score	% change in average pre‐ and post‐programme score	Average pre‐programme season score	Average post‐programme season score	% change in average pre‐ and post‐programme score	Average pre‐programme season score	Average post‐programme season score	% change in average pre‐ and post‐programme score	Average pre‐programme season score	Average post‐programme season score	% change in average pre‐ and post‐programme score
Scotland	14.5	24.0	⇧66%	8.5	10.0	⇧18%	10.0	21.5	⇧115%	13.5	20.8	⇧54%
Northern Ireland	11.0	20.5	⇧86%									
England	8.0	24.8	⇧209%	8.0	19.3	⇧141%	10.5	26.0	⇧148%	12.0	24.8	⇧106%
Wales	1.5	17.8	⇧1083%									
Republic of Ireland	7.5	21.3	⇧183%	8.0	15.0	⇧88%	9.0	19.5	⇧117%	6.5	18.3	⇧181%

Abbreviations: HDU, high‐dependency unit; ICU, intensive care unit; ROI, Republic of Ireland.

Shaded grey cells represent age group data, which could not be calculated.

Similar observations were noted in countries where age group breakdowns were available (Scotland, England and ROI), where reductions in percentage change were not noted through the scoring system. Scotland, however, did note smaller percentage increases in pre‐ and post‐ programme scores across all age groups, particularly in the <15 years and ≥65 year age group (18% and 54% percentage increases, respectively), in comparison with England (141% and 106% percentage increase, respectively) and ROI (88% and 181% percentage increase, respectively) (Table 4).

### 3.3 Mortality indicators

#### 3.3.1 EuroMOMO

Differences were not observed through the EuroMOMO model in pre‐ and post‐programme seasons between countries.

During the pre‐programme period, all countries but Wales breached the baseline threshold of 2 z‐score in the 2011/2012 season (data not available for ROI for this season). All countries breached the 2 z‐score threshold in the 2012/2013 season and did not exceed the low threshold. England and Northern Ireland also experienced greater number of weeks above the baseline threshold across these two seasons (18 and 10 weeks) in comparison with Scotland, Wales and the ROI (8, 5 and 5 weeks, respectively).

In the post‐programme period, all countries with a vaccination programme breached the 2 z‐score baseline threshold in three out of the four seasons (2014/2015, 2015/2016 and 2016/2017), with none of these countries breaching their baseline threshold in the 2013/2014 season. On the contrary, the ROI breached the baseline 2 z‐score threshold in all post‐programme seasons. All countries remained within the low impact threshold of 2 to <6 z‐score, except England, which breached the very high threshold (>16 z‐score) in 2014/2015 season and the high threshold (10 to <16 z‐score) in 2016/2017 (Figure 4).

Through the scoring system, overall reduction in percentage change between average pre‐ and post‐ programme scores was only noted for Northern Ireland and England (10% and 6% decrease, respectively); however, this was not reflected in age‐specific scores for these countries, where no reductions in percentage changes were noted (Table 5). Wales experienced no overall reduction in percentage however saw a reduction in percentage change between the average pre‐ and post‐programme scores of 50% in the <15 year of age (Table 5). Similarly, the ROI saw a reduction of 88% between the average pre‐ and post‐programme scores in the 15–64 years of age (Table 5).

**TABLE 5:** Excess mortality average z‐scores for all ages, less than 15 year olds, 15–64 years olds and 65 + years olds for pre‐ from 2011/2012 to 2016/2017 with empirical z‐score thresholds applied, UK and ROI.

Score based on the sum of weekly (week 40 to 20) thresholds across pre‐ and post‐ programme seasons	All ages			<15 years			15–64 years			65 + years		
	Average pre‐programme season score	Average post‐programme season score	% change in average pre‐ and post‐programme score	Average pre‐programme season score	Average post‐programme season score	% change in average pre‐ and post‐programme score	Average pre‐programme season score	Average post‐programme season score	% change in average pre‐ and post‐programme score	Average pre‐programme season score	Average post‐programme season score	% change in average pre‐ and post‐programme score
Scotland	4.0	4.3	⇧6%	0.0	0.8	‐	1.0	1.0	⬄0%	2.5	4.5	⇧80%
Northern Ireland	5.0	4.5	⇩10%	0.0	0.8	‐	0.5	1.0	⇧100%	3.0	4.5	⇧50%
England	9.0	8.5	⇩6%	1.0	1.3	⇧25%	4.5	4.5	⬄0%	7.0	8.3	⇧18%
Wales	2.5	2.8	⇧10%	0.5	0.3	⇩50%	1.5	1.5	⬄0%	2.0	2.0	⬄0%
Republic of Ireland	5.0	5.0	⬄0%	1.0	1.0	⬄0%	2.0	0.3	⇩88%	6.0	6.5	⇧8%

Abbreviation: ROI, Republic of Ireland.

#### 3.3.2 FluMOMO

Differences between countries who were vaccinating all primary school‐aged children and those who are incrementally vaccinating primary school‐aged children were noted through the influenza‐attributable mortality model, FluMOMO.

During the pre‐programme period, little influenza‐attributable deaths were noted across all countries in 2011/2012; however, all countries experienced influenza deaths above their respective baselines in the 2012/2013 season (Figure 6). Cumulative influenza‐attributable mortality rates for all ages also remained similar across all countries with the rate for the ROI starting at a lower rate in comparison to other countries (Figure 7A).

**FIGURE 5:**
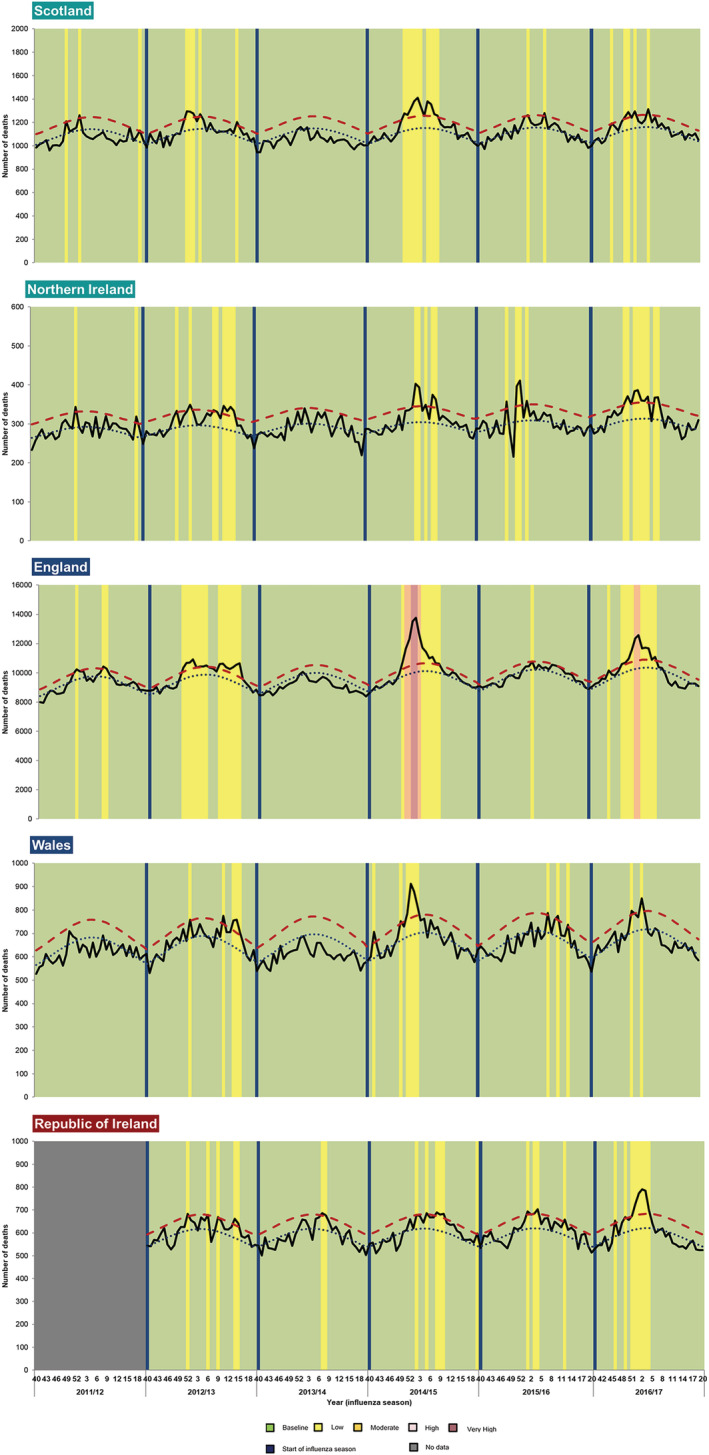
Weekly observed and expected number of all‐cause deaths (EuroMOMO) in all ages from 2011/2012 to 2016/2017 with empirical z‐score thresholds applied, UK and Republic of Ireland (ROI).

**FIGURE 6:**
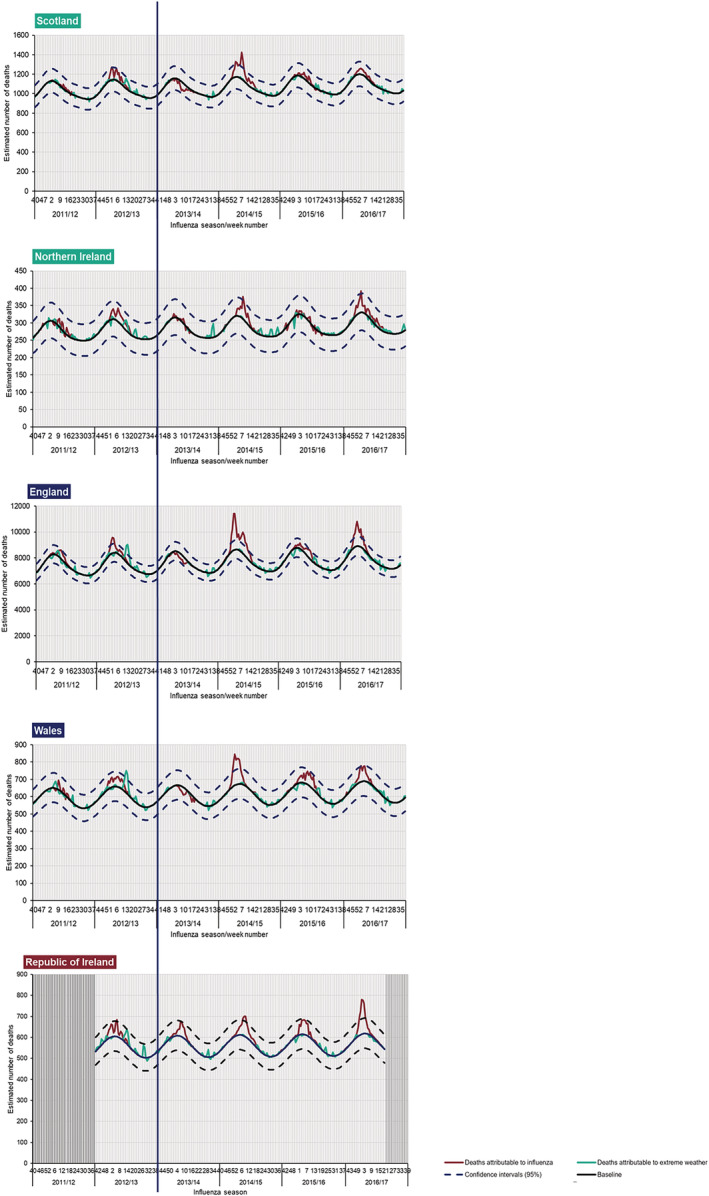
Weekly estimated number of influenza‐attributable deaths (FluMOMO) in all ages, from 2011/2012 to 2016/2017, UK and Republic of Ireland (ROI).

**FIGURE 7:**
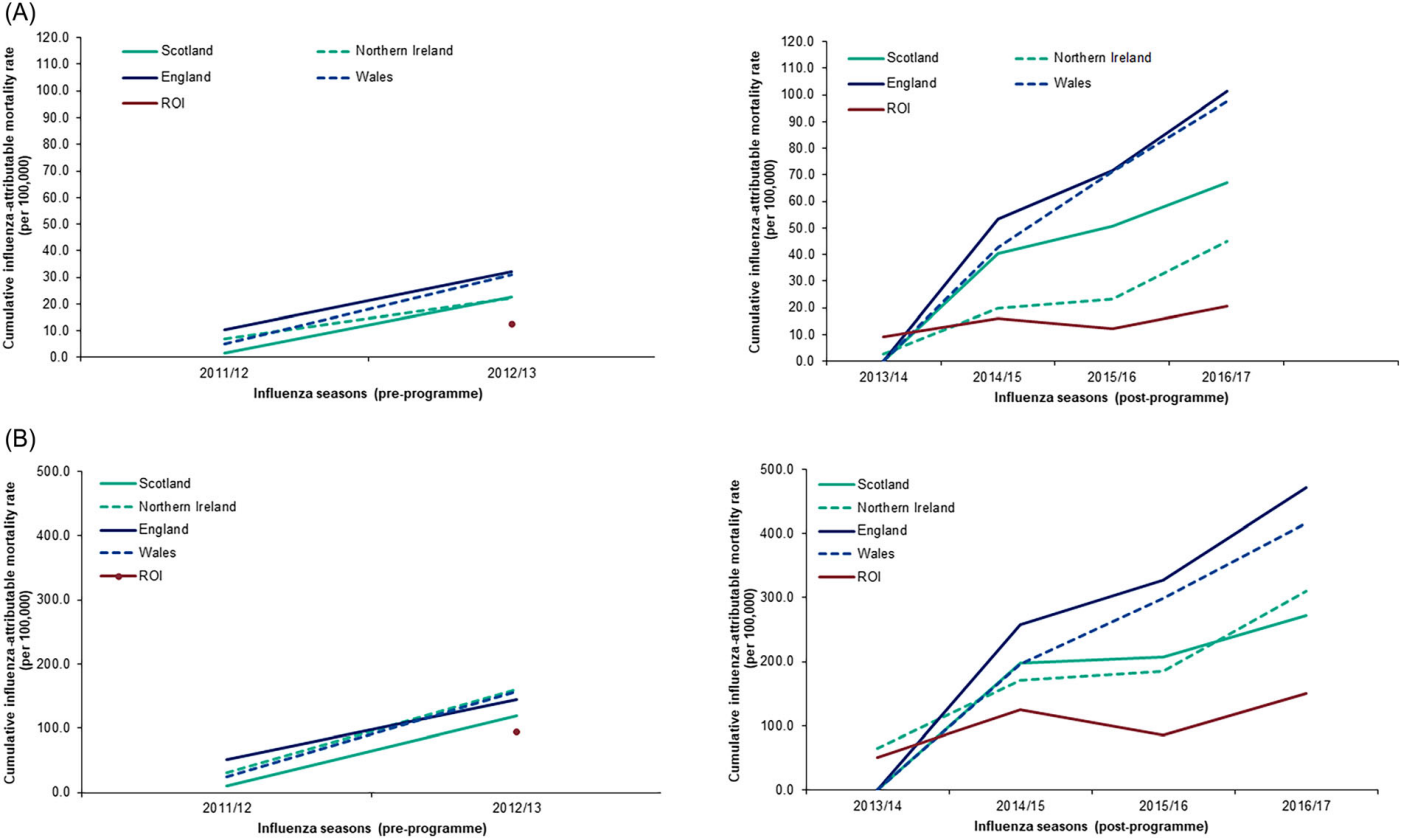
Cumulative influenza‐attributable mortality rate per 100,000 population for pre‐ and post‐programme in (A) all ages and (B) 65 + years, UK countries and Republic of Ireland.

The post‐programme period highlighted that England, Wales and the ROI experienced influenza‐attributable deaths above their respective baselines and exceeding their 95% confidence intervals, in the 2014/2015, 2015/2016 and 2016/2017 seasons (Figure 5). In comparison, Northern Ireland experienced influenza‐attributable deaths above its respective baselines and exceeding its 95% confidence intervals in the 2014/2015 and 2016/2017 seasons and Scotland only in the 2014/2015 season. In the 2015/2016 season, fewer influenza‐attributable deaths were noted amongst countries vaccinating all school age children (Scotland and Northern Ireland) in comparison with those observed in countries incrementally vaccinating school children (England and Wales) (Figure 6). Comparisons of cumulative influenza‐attributable mortality rates for all ages between countries in the post‐programme period highlighted lower cumulative rates experienced in Scotland and Northern Ireland and the ROI (67.1, 45.0 and 20.6 per 100,000, respectively) than in England and Wales (101.4 and 97.5 per 100,000, respectively) (Figure 7A). A similar observation was also noted in those aged ≥65 years (Figure 7B).

## 4 DISCUSSION

This study assessed the impact of the paediatric LAIV programme in the UK, based on the differential roll‐out of the programme across the UK and the ROI that did not have a universal paediatric programme, comparing observations from influenza surveillance in primary and secondary care and mortality indicators, from the 2010/2011 season to the 2016/2017 season. Overall, our findings highlight those countries who were vaccinating all primary school age children (Scotland and Northern Ireland), observed reductions in overall GP ILI consultations and influenza‐attributable mortality rates, in comparison to countries who were incrementally vaccinating (England and Wales) or not vaccinating primary school age children (ROI). Although significant reductions were not seen through the secondary care indicator across all countries, vaccinating countries still observed lower percentage increases in influenza‐related ICU admissions than partially/non‐vaccinating countries.

To better understand our findings, it is significant to note that since the introduction of the paediatric influenza vaccine programme in the UK, Scotland and Northern Ireland have seen the highest uptake amongst their primary school‐aged children, with the average LAIV uptake being 72.6% and 77.2%, respectively, between 2015/2016 and 2017/2018.^23^ This compares to an average uptake of 56.2% and 66.2% in England and Wales.^23^ Our study findings therefore suggest that countries with higher vaccine uptake levels across all cohorts in primary school age children have observed greater reductions in primary care ILI consultation rates as well as fewer influenza‐attributable deaths in the subsequent seasons after the introduction of the programme.

Reductions were observed overall (all ages) and in the <15 years age group primary care ILI consultation rates across all countries between pre‐ and post‐programme seasons with the greater reductions seen amongst countries vaccinating all primary school age children at high uptake levels, highlighting both the indirect and the direct impact of the programme. These findings are concurrent with previous studies where significant reductions in primary care consultations amongst vaccinating areas compared to non‐vaccinating areas/seasons in individual countries have been observed.^5^, ^6^, ^7^, ^8^, ^9^ Indirect impacts were also observed amongst the 65 + years age group with all vaccinating countries noting reductions in primary care ILI consultations in this group. On the contrary, the ROI noted an increase of 95% in this age group between the pre‐ and post‐ programme seasons, which may be explained by the very high primary care ILI consultations reported in this age group in the post‐ programme seasons.^24^ This reinforces the need to monitor impact of targeted vaccination programmes across all age groups but also highlights the indirect impact of the paediatric programme on other age groups.

The absence of reductions in overall secondary care ICU admissions in the pre‐ and post‐programme seasons has also been noted in previous studies where no or very little impact had been observed. This has been associated with seasonal influenza vaccine strain and circulating strain mismatch.^7^, ^8^, ^25^, ^26^ This could be a potential explanation of our findings where two of the post‐programme seasons (2014/2015 and 2016/2017) observed suboptimal matching of the influenza A(H3) subtype as well as being dominated by circulation of A(H3), which is known to affect the older population more adversely. The increasing and wider use of rapid point‐of‐care testing in secondary care may have also contributed to an increased detection in influenza particularly in the most recent seasons of the study.^27^ Despite the apparent reduced impact against severe disease, smaller percentage increases in ICU influenza admissions were noted amongst fully vaccinating countries in the post‐programme seasons in our findings, suggesting a likely impact effect.

Our findings of little to no impact in all‐cause excess mortality regardless of the countries' influenza vaccine programmes are contrary to those observed in previous studies.^28^, ^29^, ^30^ Reichert et al. found that influenza vaccination programmes targeted at school children in Japan and the United States prevented an average of 43,000 all‐cause deaths per year.^29^ A later study found an indirect impact of the Japanese paediatric vaccine programme as a reduction in deaths amongst Japan's older population.^30^ It is important to note as per our findings amongst secondary care indicators the contribution of vaccine mismatch, and the circulating virus can also be a contributing factor to the lesser impact on all‐cause mortality. Our finding, however of decreased cumulative influenza‐attributable mortality rates in the post‐programme period, overall and in those aged ≥65 years, amongst vaccinating countries in comparison with partially vaccinating countries is encouraging and highlights a likely impact against severe disease. The use of a method such as the FluMOMO model to highlight the contribution of different possible causes of all‐cause excess is important when introducing new vaccination programmes. Our findings highlight that excess mortality may however also be related to a range of factors besides influenza, including systematic differences in the recording of these data, the contribution of other respiratory infections, winter pressures on health services and cold weather.

The strengths of this study include the use of standardisation methods to allow comparisons between countries. The MEM method has been analysed and adopted in the UK to be a better approach in reporting and assessing the impact on healthcare services during seasonal influenza periods.^17^ This method has also been adopted by several European countries and has become part of a wider WHO initiative.^11^ The EuroMOMO model is used by a network of approximately 26 European countries analysing and reporting all‐cause excess mortality weekly.^14^ Additionally, we introduce a new method of quantifying thresholds to compute scores to allow comparisons across different time periods (pre‐ vs. post‐programme scores), which can be adapted at an individual country basis. Another strength is the use of national population‐level data, which has allowed us to better analyse the impact of vaccine programmes in all countries. For example, information on all influenza ICU admissions across each respective countries was available for the duration of the study, and population denominators were used. Similarly, national death registration data from each country have been used, and excess mortality rates were calculated based on population denominators.

There are some limitations that need to be considered, as although we have tried to address differences between surveillance schemes in the countries by using standardised methods, underlying differences in factors such as case definitions, access to care and health‐seeking behaviours between countries remain. For example, some countries' case definitions for the secondary care indicator included both ICU and HDU admissions, whereas others only included ICU admissions (Scotland and ROI). Changes in laboratory testing overtime in secondary care with the introduction of rapid point‐of‐care testing may have increased sensitivity in these systems, which could explain the increases shown through our findings across all countries.

In conclusion, the findings of this study are overall encouraging and support the ongoing implementation of universal paediatric influenza vaccination programmes and their continuation. It has provided an important insight into the impact of such a programme on countries at different time points in their roll‐out as well as reinstating the reductions of the burden on primary care consultations and highlighting evidence of reductions in influenza‐attributable deaths. Further work is required to understand the impact of vaccination on other secondary care and all‐cause excess mortality severity indicators.

## AUTHOR CONTRIBUTIONS

**Mary A. Sinnathamby:** Data curation; formal analysis; methodology; visualization; writing – original draft. **Fiona Warburton:** Data curation; formal analysis; methodology; writing – review and editing. **Arlene J. Reynolds:** Data curation; writing – review and editing. **Simon Cottrell:** Data curation; writing – review and editing. **Mark O'Doherty:** Data curation; writing – review and editing. **Lisa Domegan:** Data curation; writing – review and editing. **Joan O'Donnell:** Writing – review and editing. **Jillian Johnston:** Writing – review and editing. **Ivelina Yonova:** Data curation; writing – review and editing. **Suzanne Elgohari:** Data curation; writing – review and editing. **Nicola L. Boddington:** Data curation; writing – review and editing. **Nick Andrews:** Conceptualization; methodology; writing – review and editing. **Joanna Ellis:** Data curation; writing – review and editing. **Simon de Lusignan:** Data curation; writing – review and editing. **Jim McMenamin:** Conceptualization; writing – review and editing. **Richard G. Pebody:** Conceptualization; supervision; writing – review and editing.

## CONFLICT OF INTEREST STATEMENT

Simon de Lusignan has received funding through his university for vaccine‐related research from AstraZeneca, GSK, Sanofi, Seqirus and Takeda and has been a member of advisory boards for AstraZeneca, Sanofi and Seqirus. No conflicts of interest are declared for the other authors.

### PEER REVIEW

The peer review history for this article is available at https://publons.com/publon/10.1111/irv.13099.

## Data Availability

Applications for requests to access relevant anonymised data should be submitted to UK Health Security Agency (UKHSA) using this form: https://assets.publishing.service.gov.uk/government/uploads/system/uploads/attachment_data/file/1064456/2022-01-01-UKHSA-Data-Application-Form-Primary-Applicant_-_gateway_v0.3.pdf.
